# Supplementary material for: Inhibition of the sodium-dependent HCO3- transporter SLC4A4, produces a cystic fibrosis-like airway disease phenotype
Source: eLife. 2022 May 30;11:e75871. doi: 10.7554/eLife.75871 (PMC9173743; doi:10.7554/eLife.75871)
Supplement: Supplementary file 1. [file elife-75871-supp1.docx]

Supplementary Table 1, Primers for hBECs.

| Gene name | Alias/Isoform | Gene bank access number | Primer sequence 5>3 | Amplicon size (bp) |
| --- | --- | --- | --- | --- |
| *SLC4A4* | NBCe1-A | NM_003759.4 | Forward: GATTTGGGAGGCTTAGCAGGA  Reverse: GGCTTCCCTTCCACATTTTCAG | 53 |
| *SLC4A4* | NBCe1-B/C | NM_001098484.3 | Forward: AGGAGGATGGAGGATGAAGCT  Reverse: ACTCTTCGGCACATGGACTC | 120 |
| *SLC4A5* | NBCe2 | NM_021196.3 | Forward: CAGCCATGCATGAAATCGGG  Reverse: CAAAGGTGATCGCGTTGGTG | 183 |
| *SLC4A7* | NBCn1/NBC2 | NM_003615.5 | Forward: CTGCAGGCTCAAGGTGTACA  Reverse: CTGACGCTGACTCTCTTGGG | 110 |
| *SLC4A8* | NDCBE | NM_001039960.3 | Forward: TGTATTGGACTGTGGACCGC  Reverse: GCATGTGGATGGGGTAGGTC | 181 |
| *SLC4A10* | NBCn2 | NM_001178015.2 | Forward: CCTCCACACCAGCAAGAGAG  Reverse: TTGGGATTGGGACTTCAGCC | 178 |
