# Supplementary material for: Inhibition of the sodium-dependent HCO3- transporter SLC4A4, produces a cystic fibrosis-like airway disease phenotype
Source: eLife. 2022 May 30;11:e75871. doi: 10.7554/eLife.75871 (PMC9173743; doi:10.7554/eLife.75871)
Supplement: Supplementary file 2. [file elife-75871-supp2.docx]

Supplementary Table 2, Primers for mouse.

| Gene | Accession number | Primer sequence 5>3 | Annealing T | Amplicon size |
| --- | --- | --- | --- | --- |
| *Slc4a4* | NM_018760.2 | Forward: TCCCTTCATTGCCTTTGTTC  Reverse: CAAGGTGGCGATAGCTCTTC | 54.2 C° | 151 bp |
| *Slc4a5* | NM_001166067.1 | Forward: TGAACACAACCACGGTCAAT  Reverse: CGTAGCTCAGGCACTCCTTC | 54.2 C° | 126 bp |
| *Slc4a7* | NM_001033270.2 | Forward: CGCATAGAGCCTCCAAAAAG  Reverse: GCATGGTGATCATCCTCCTT | 54.2 C° | 131 bp |
| *Slc4a8* | NM_021530.2 | Forward: GGGCAGCAGTACCATGAGAT  Reverse: GTCCAGGAACTCGTCAATCC | 54.2 C° | 126 bp |
| *Slc4a10* | NM_001242378.1 | Forward: TTCAAGACCAGCCGCTATTT  Reverse: GGATCCCAATGGCATAGTCA | 54.2 C° | 109 bp |
| *Slc4a4*-A | HQ018820.1 | Forward: CTCAGGGTTTTCCAGCCAATG  Reverse: TCATCGAGTTCCGTGAAGAGC | 59.7 C° | 137 bp |
| *Slc4a4*-B | NM_018760.2 | Forward: AGAAGGTCACCACACGATCTACATTG  Reverse: GTTTGAGGATGCTGCTGCTG | 57.9 C° | 179 bp |
